# Wetting/Drying Behavior of Lime and Alkaline Activation Stabilized Marine Clay Reinforced with Modified Coir Fiber

**DOI:** 10.3390/ma13122753

**Published:** 2020-06-17

**Authors:** Fatin Amirah Kamaruddin, Vivi Anggraini, Bujang Kim Huat, Haslinda Nahazanan

**Affiliations:** 1 Department of Civil Engineering, Faculty of Engineering, Universiti Putra Malaysia, UPM Serdang, Selangor 43400, Malaysia; fateen_58@yahoo.com; 2 Civil Engineering Discipline, School of Engineering, Monash University Malaysia, Bandar Sunway, Selangor 47500, Malaysia; vivi.anggraini@monash.edu; 3 Department of Engineering, Universiti Putra Malaysia (Bintulu Campus), P.O Box 396, Nyabau Road, Bintulu, Sarawak 97008, Malaysia; bujangkh2001@upm.edu.my

**Keywords:** wetting/drying cycles, durability, curing periods, lime, alkaline activation, compressive strength

## Abstract

The durability of natural and treated clay soil stabilized with lime and alkaline activation (AA) affected by environmental factors (hot and humid) was determined in this study. Investigation and evaluation on the strength of the soil, moisture content, and volume change of the specimen were determined at each curing period (7, 28, and 90 days) based on the weather conditions. An unconfined compressive strength (UCS) of the specimen at three different wetting/drying cycles (one, three, and five cycles) was determined. The findings show that the strength of the treated specimens fluctuated with increment and decrement strength (one and three cycles) in the range of 1.41 to 1.88 MPa (lime) and 2.64 to 8.29 MPa (AA), while for five cycles with a curing period of 90 days the decrement was in the range of 1.62 to 1.25 MPa and 6.06 to 5.89 MPa for lime and AA, respectively. The decrement percentage for treated samples that were subjected to five cycles of wetting and drying in 90 days was found to be 20.38% (lime) and 38.64% (AA), respectively. Therefore, it can be summarized that wetting/drying cycles have a significant influence on the durability, strength, and the volume changes of the specimens.

## 1. Introduction

Marine clays are natural sediments that originate from coastal areas under saline environmental conditions [1]. They tend to have a high percentage of water content, low permeability, low shear strength, and high compressibility which causes instability, according to Jiang et al. [2,3,4]. Treatment on soil along with the inclusion of a reinforcement element is one of the solutions that could lead to a better performance of soil. Materials are important for the improvement of the soil.

Stabilization using lime is one of the common techniques in improving the earthworks as its usage is continuous since its introduction in the past century. The modification using lime has been widely used due to the rapid effectiveness in subgrade soft soil and building embankment that changes the geotechnical characteristic, such as the soil compaction, shear strength, and plastic limit. Past researches [5,6] have proven that the addition of lime in the soil also increased the performance towards curing time, improved the reduction of soil plasticity, increased the mechanical properties of soil, and improved the workability.

Fly ash (FA) is one of the binders used for stabilization and is a waste by-product that has been used as precursors in an alkaline activation (AA). The utilization of fly ash as admixture or filler material is more conducive as it is not only a more sustainable and environmentally friendly waste material, but it can also be promoted for land conservation purposes. There are many benefits of FA as a precursor of soil treatment, such as exhibiting higher mechanical strength, more ductility, and a decrease in the collapsible potential of the soil [7,8,9]. Hence, the engagement of waste material in AA that leads to sustainable and environmentally friendly materials is more pleasant for future development.

Nevertheless, [10,11] noted that treating the soil with lime and alkaline activation binders displays a significant post-peak in the compressive strength where the result shows the brittleness on the soil. Limitation on the post-peak of the soil is important in order to be able to anticipate expected seismic loads or high lateral earth pressures that might occur in the future [12,13]. Researches by [8,14,15,16] state in their findings that when fiber is added to the soil matrix that is known for having high tensile strength, the shear stress that is generated while the force is applied between the soil particles is transferred to the fibers to form the tensile strength on the soil, thus enhancing the strength between soil and fiber and therefore improving the soil brittleness to ductile post-peak behavior. This has been supported by [17,18] with their findings on the inclusion of treated fiber in soil where the compressive strength of the soil improved significantly compared to untreated soil. Meanwhile, the selection of a proper fiber is important as it could help in sustaining the tensile strength for the soil.

Fiber can be classified into two types that are either natural fiber or synthetic fiber where each type of fiber has its own specialty that differs from others. Coir fiber is a type of natural fiber. The selection of coir fiber is also due to the awareness that it has an environmentally friendly and sustainable life. Other than that, the usage of coir fiber as a reinforcement gains interest in research studies due to its advantages such as being abundantly available, of low cost, a renewable resource, lightweight, and having good mechanical properties compared to other natural fibers [19,20,21,22]. Coir fiber as a soil reinforcement is not a new development as this material is widely used in many ways in soil improvement. The natural fiber is composed of biopolymers that have high lignin, cellulose, and hemicellulose which have good connection with the chemical, biological, and mechanical properties of the fiber [23,24]. Of all of the types of fiber, coir fiber has the highest lignin content which contributes to having a low degradation of fibers, rigidity, and high strength retention compared to other natural fibers [25,26]. Despite that, coir fiber also has several disadvantages such as high moisture absorption which lead to poor interfacial bonding, increasing the rate, and accelerating the biodegradation of fiber [27,28,29,30,31]. To overcome these problems, improvement on the fiber surface is needed either with the chemical or physical coatings on the fiber, where this method is found to be the most efficient. By [32], this method is suitable as it could prevent moisture from going into the fiber as it enhances the fiber strength and becomes more durable in an alkaline environment. Findings by [23,33,34] reported that the method has decreased the water absorption as well as increased the stiffness of the fiber. Mechanical properties show a significant improvement for the coir fiber as it is being achieved through the chemical treatment on the fiber surface, thus promoting the bonding between the fiber matrix [35,36]. Literature on supporting the advantages of treated fiber as soil reinforcement in the soil are well established. However, it is noticed that there is a lack of investigation on the effectiveness of the inclusion of fiber in soil admixture in terms of the effect of durability on the compressive strength of soil. The investigation on the effect of durability on soil strength is critically needed for the soil before considering it as a ground improvement method.

Therefore, this research attempts to study the effectiveness of the durability test for the treated soil (lime and AA) with the inclusion of fiber subjected to wetting drying cycle tests. The changes on strength and physical properties for the both treated clay soils were examined by conducting the unconfined compressive strength test (UCS). For the microstructural changes, scanning of electron microscopy analyses (FESEM) and Energy Dispersion X-ray (EDX) were performed to explain the reaction and strength of the clay soil development.

## 2. Materials and Methods

### 2.1. Materials Used

#### 2.1.1. Soils

**Figure 1:**
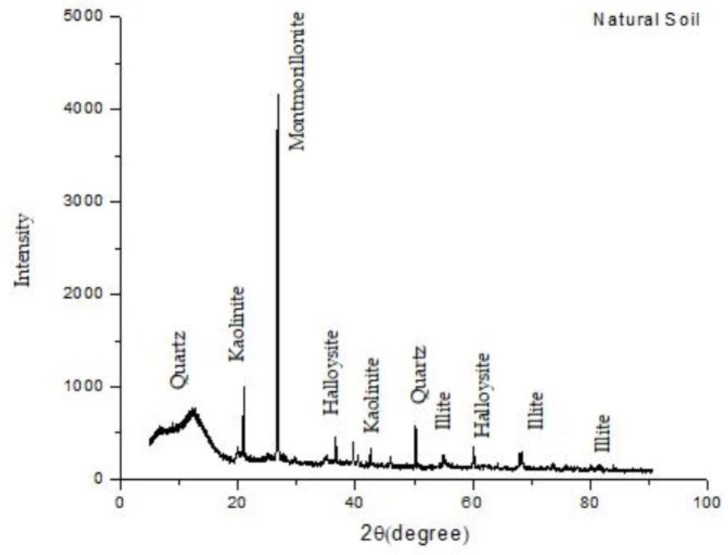
XRD pattern for marine clay soil.

**Figure 2:**
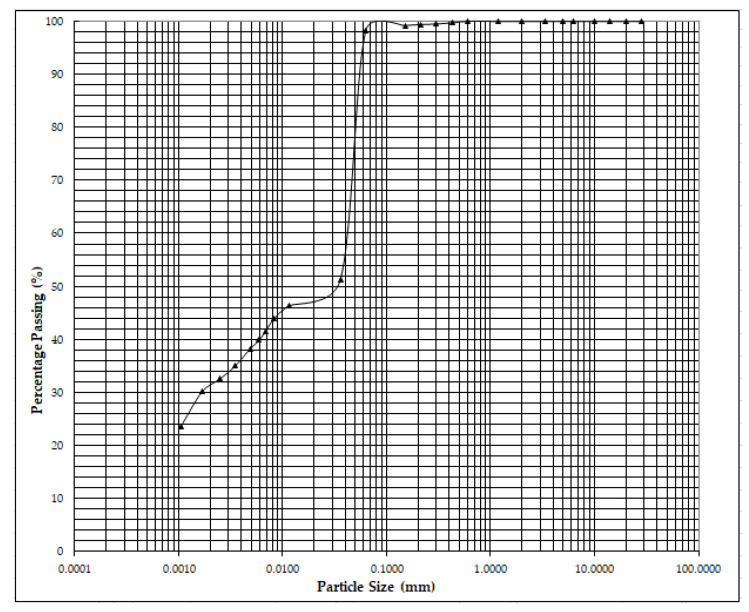
Marine clay soil distribution curve.

The soil used in this study is a clay soil collected from a coastal area in Klang, Selangor. The physical properties of the marine clay were tested in accordance to BS 1377-2-1990 [37] and the results are presented in Table 1. The mineralogy composition of the clay soil was obtained by conducting X-Ray diffraction analysis, revealing high contents of montmorillonite, illite, quartz, kaolinite, and halloysite (Figure 1). According to the Unified Soil Classification System (USCS), the soil specimen was categorized as clay with a high plasticity of sand (CH). Figure 2 shows the grain distribution curve for marine clay soil.

#### 2.1.2. Coir Fiber

Coir fiber used in this study was obtained from a factory at Batu Pahat, Johor. A total of 1% fiber was selected as the most effective percentage for soil reinforcement to improve the ductile behavior of the treated soil as suggested by [38]. Research by [38,39,40,41] suggest that coir fiber with more than 1% will reduce the soil strength as the fiber will contribute in forming lumps and could not appropriately be mixed with the soil admixture. The fibers with a diameter size of 0.18 to 0.35 mm and a length of 1 cm each were prepared before being treated with a calcium chloride solution (Anggraini [38]). Calcium chloride was selected to treat the fiber as it shows the most effective results in enhancing the compressive strength compared to other solutions [42,43]. Table 2 summarizes the properties of coir fiber used in this study.

#### 2.1.3. Lime

Hydrated lime (Ca(OH)_2_) was used in this study as a binder for the stabilized soil. The chemical was supplied by Evergreen Engineering, which has a chemical content of (lime) more than 90% applicable for this study. A total of 5% of lime was used as stabilizer, which is considered to have a positive effect as lime fixation point and particularly succeeds in generating maximum strength for the workability of the soil [40,44].

#### 2.1.4. Fly Ash

Fly ash (FA) is a material that originates from a bituminous power plant coal combustion obtained from a local factory near Selangor. The type of FA used in this study was class F type, which was classified as having low calcium content ASTM C 618 [45]. The cumulative percentage of SiO_2_, Al_2_O_3_, and Fe_2_O_3_ as demonstrated in Table 3 shows that FA contains a chemical composition of more than 70%, fulfilling the standard requirements in the ASTM C 618 [45] for Class F type. The chemical composition of fly ash was done by the XRF test.

#### 2.1.5. Activator

Activators play an important role in the development of soil strength. In this study, potassium hydroxide (KOH) containing K^+^ cation was selected as the alkaline activator that is suitable in mixing with fly ash. Generally, the selection of a suitable concentration for the activators is between 4.5 and 18 molar [46,47]. Molarity with a concentration of 15 mol and above has been reported as a non-viable due to the viscosity of the solution which leads to an increase of the parent soil, and thus leads to poor unconfined compressive strength (UCS) results and a poor condition of the semi-plastic mixture [46]. Pourakbar et al. [46] also stated that, although 10 mol and 12.5 mol display results of similar strength, the usage of 10 mol of KOH is the most effective concentration for soil improvement in terms of economy and practicality. Therefore, 10 mol of KOH was selected to be used for this study as it is the most effective concentration to be mixed with soil. The solution was prepared by dissolving the KOH in one liter of distilled water. The weight of the KOH pellets molecular was 56.105 g/mol, thus 561.05 g is needed to make 10 mol of solution. After the concentration diluted, the solution was left to cool down to ambient temperature since the reaction is strongly exothermic, as with all strong bases [48].

### 2.2. Method

#### 2.2.1. Sample Preparation

The collected soil sample was dried in an oven at a temperature of 105 °C for 24 h before being crushed and sieved passing through a 2 mm sieve. In this study, four types of main material were used, which are soil, hydrated lime, fly ash, and coir fiber. All of the specimens were prepared by using a hand mixer. As for soil stabilized with lime, 5% of hydrated lime (15 g out of the total dry soil weight) and 1% of treated fiber (3 g out of the total dry soil weight) were used in order to increase the strength of soil (Anggraini [38]). For AA, 60% of FA and 1% of treated coir fiber were used for the designation of the mixture as suggested by [48]. The soil mixtures for both treatments (lime and AA) were done with an addition of distilled water (for lime stabilizer) and alkaline activator solution (for AA stabilizer) at an optimum moisture content. The mixture was mixed thoroughly until it achieved a uniform blend. The designation mix for this study is presented in Table 4.

#### 2.2.2. Experimental Procedure

##### Unconfined Compression Strength Test

The evaluation strength of untreated and treated clay soil specimens was determined by using an unconfined compression test. The specimen was tested according to BS1377-7-1990 [49]. Each sample was tested until it reached the failure axial strain of 20%. Three duplicate samples were tested for each specimen in order to have an average result for the UCS test. The sample was prepared by dividing it into three layers in the cylindrical mold with a height of 100 mm and a diameter of 50 mm using a 45 mm diameter steel rod. Each of the layers was tamped with 27 blows. The compacted specimen was adapted based on the recommendation BS1377-7-1990; ASTM D 2166 [4,49,50]. The sample was then extruded carefully and wrapped with a plastic cover and aluminum foil in order to prevent loss of moisture content. Soil specimens were then stored in a curing chamber for 7, 28, and 90 curing days. The selection of the curing periods for the sample were based on the short- and long-term performances for the specimens [51]. After the curing date was achieved, the sample was then continued with the durability test.

##### Durability Test

The wetting/drying cycles conducted in this study were in accordance with the ASTM D 599 [52,53,54,55]. After the curing periods of 7, 28, and 90 days completed, the specimens were air-dried for 24 h before being submerged for the following 24 h. In [56], one cycle of wetting/drying is represented by completing both the drying and the submerging process that takes 48 h. In this study, 3 numbers of cycles were employed, which were on 1, 3, and 5 cycles. Table 4 shows the mixture proportion used in this study (wetting/drying test).

##### Durability Index, Dry Density, Moisture Content, and Weight Loss

In this section, the determination of the durability index, dry density (ρd), moisture content (MC), and weight loss is for the untreated clay and treated clay with lime and alkaline activation, according to [52] and BS 1377-1990 [37]. The determination of this test is related to the effect of the soil behavior which was subjected to the wetting/drying cycles. The calculation of the durability index for the specimens was performed based on the peak compressive strength (*qu*) of the soil specimen at each cycle (1, 3, and 5) towards the peak compressive strength (*qu*) of the specimen at the none number of the cycle (without wetting/drying cycles) at different curing periods.

The dry density of the soil is determined as the mass of the dry soil contained in the unit volume of the undried materials where the calculation is based on Equation (1) below [37].

(1) $$\rho_{d}=\frac{\left(100+\rho \right)}{100+w}.$$

As for the moisture content of soil, the determination is based on the percentage of dry mass of the soil. For this test, the weight of the soil in wet and dry conditions was recorded and calculated based on Equation (2).

(2) $$w=\frac{m_{2}-m_{3}}{m_{3}}\times 100\;\left(\%\right),$$

where ρ is the bulk density of the soil specimen and *w* is the water/moisture content of the soil specimen. m_2_ represents the soil specimen in wet condition and m_3_ represents the soil specimen in dry condition.

Next, the parameter of the weight of soil loss is expressed as the initial dry unit of treated soil specimens after being subjected to wetting/drying cycles over the untreated soil specimens [57].

#### 2.2.3. Microstructural Analysis

The FESEM and EDX analyses were conducted to analyze the morphology on the soil surface and the element composition of the untreated and treated soil specimen using the dispersive X-ray spectrometer The dry specimen was mounted on the platinum sample with adhesive carbon tape before being sputter-coated using the Q150R S Sputter coater (Quorum, Quorum Technologies Ltd, East Sussex, UK). The sputter coater is used to improve the surface electrical conductivity and thus reduces the charging effect. The micrographs of the specimen were obtained and reviewed under SU 8010 FESEM (Hitachi SU8010, Oxford-Horiba Inca XMax50, Hitachi High- Technologies, Tokyo, Japan).

## 3. Results

### 3.1. Effect of Wetting and Drying Cycles on Soil Strength

**Figure 3:**
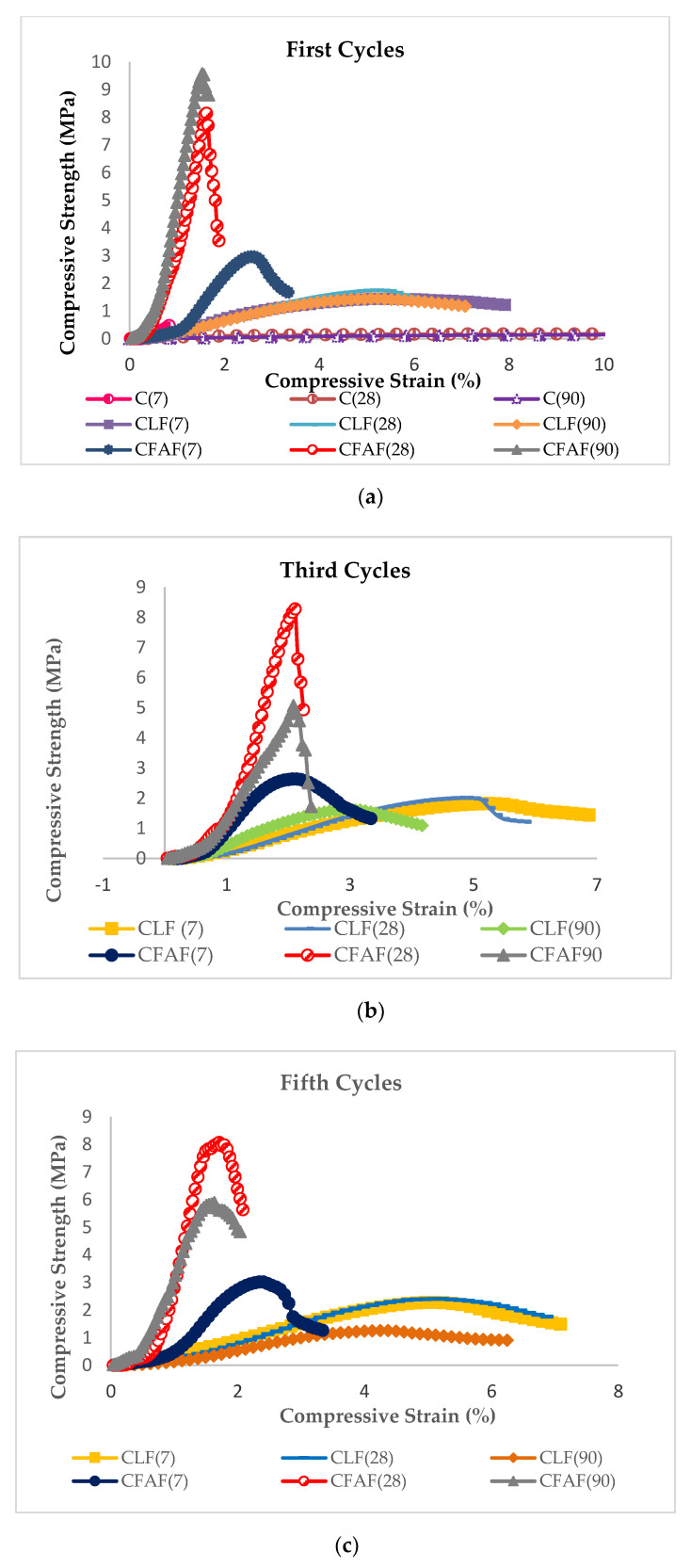
(**a**), (**b**), and (**c**) Compressive strength against compressive strain for 1, 3, and 5 cycles.

The relationship between the compressive stress versus compressive strain of the untreated clay and treated clay with lime and AA subjected to wetting/drying cycles after 7, 28, and 90 curing days is presented in Figure 3a–c respectively. In general, as can be seen in Figure 3, the result of the compressive strength is fluctuating (increasing and decreasing), influenced by the increase of the number of cycles for untreated and treated specimens at different curing periods. It is notable that, with the inclusion of the fiber in the stabilized soil, the reaction of the stress–strain curves changes from brittle to ductile behavior, as shown in Figure 3. For the untreated soil specimens, the results show that no significant decrement or increment occurred in the different curing periods. The measurement of the strength for the untreated clay specimen at different curing periods is performed in order to compare it with the result of the treated specimens at different curing periods although the increment is small. The untreated clay specimens could only resist the first wetting/drying cycle and started to break at the end of the second cycle. It can be explained that without soil stabilization, the clay soil shows a weakness and inability to withstand the load and is not suitable with mainly wet and dry weather. The findings of this current study are consistent with those of past research by [38,53] on the untreated clay soil. Therefore, treatment on the untreated soil is one of the alternatives or solutions that are able to increase the strength as well as the ability to resist the wetting/drying cycles.

Next, the wetting/drying cycles have a large effect on the increment of strength for the lime treated sample (for one and three cycles) in the range of 1.41 to 1.88 MPa and 2.64 to 8.29 MPa. While for the five cycles (Figure 3c) of a period of 90 curing days the decrement was in the range of 1.62 to 1.25 MPa and 6.06 to 5.89 MPa for lime and AA, respectively. As for the AA treated soil, there is a sharp decrement on the first three cycles for the 90 days of the curing period which can be seen in Figure 3a,b. This may be due to the chemical reaction (ion) that occurred inside the soil, which contributes to the decrement of the soil strength. The percentage of the decrement on the strength of the treated samples (lime and AA) subjected to five cycles of wetting/drying for 90 days was found to be 20.38% and 38.64%, respectively. Although the decrement percentage is higher in the treated samples with AA, the sample treated is still better than the specimen treated with lime. This behavior explains that the AA treatment towards the soil specimen increases the interfacial bonding due to the chemical reaction (before the wetting/drying cycles) occurring between the soil particles compared to lime and untreated soil specimens. The bonding reaction between the soil particles and stabilizer also increases the strength with increasing curing periods.

**Figure 4:**
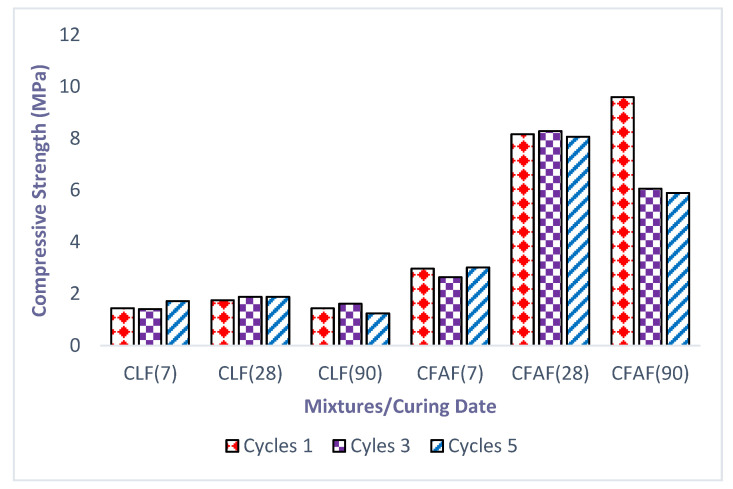
Evolution strength for all specimens.

In addition, the soil stabilized with AA reached the highest approximate dry state compared to the lime-stabilized soil. The high dry state of the specimen is due to the tendency of the stabilizer to fill the pores between the soil particles, thus sealing the voids and presenting water to infiltrate into the soil. Furthermore, based on Figure 4, the effect of the dry soil state for the wetting/drying cycles on the treated specimens shows significant results, although there is a fluctuation in increment or decrement over the curing period, where the results are significantly higher between the cycles which can be attributed to the untreated specimens. This is due to the condition where in the wet stage, the voids in the soils are already filled with water and the tendency to absorb water is therefore limited [52]. Reference [52] also stated that submerging the specimen from a dry state to a wet state has a de-accelerating effect since it is interrupting the chemical reaction that occurs between the soil and the stabilizer. The bonding that develops between the soil particles and the stabilizer will also decrease with the increase of wetting/drying cycles.

Other than that, the wetting/drying cycles show an increase in compressive strength after the first three cycles for 7, 28, and 90 days of treated soil and start to decrease at five cycles only at 90 days of the curing period. The increase of the compressive strength on the curing period as well as the cycles might be due to the low absorption of water towards the soil specimen. In addition, the strength increment of the soil specimen is likely to show a high maturity of the curing period for the specimen where in this case, the specimen able to withstand the effect of the wetting/drying as the soil particles start to strengthen together. The deterioration of the strength for the treated soil after three cycles (for 90 days) might be due to the bond and the interlocking between the soil particles becoming loose.

However, the reduction on the strength from the third cycle onward is observed as not being too significant. Not to mention that the particles in the specimens are going through disruption which then causes the changes of the structure in the particles. The authors of [52] show similar findings related to the chemical reactions that were completed right after the third cycle which affected the acceleration of the soil strength. The reaction caused the bond between the soil particles and stabilizer to increase in strength with the increase in curing periods. It can be concluded that the usage of a stabilizer in the untreated clay soil has clearly resulted in strengthening the specimen during the curing periods. This can be attributed to the chemical reaction that strengthens and hardens the soil with fiber embedded in the soil, which causes better interlocking between the soil particles. The statement was supported by several past researchers where the inclusion of fiber in the soil matrix would be able to help the shear stress generated between the soil particles that are then transferred to the fiber which is known for having high tensile strength [15,16,55].

### 3.2. Effect of the Durability Index, Wetting/Drying Cycles on Dry Density, Moisture Content, and Weight Loss Percentage

**Figure 5:**
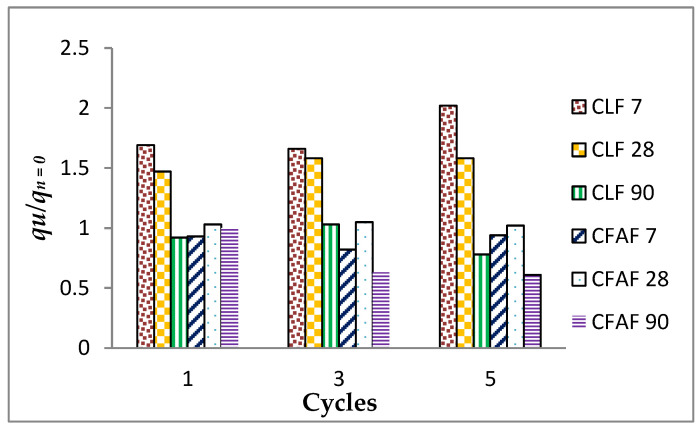
Durability index versus cycles.

For a better understanding regarding the effect of the wetting/drying cycles to the strength performance of the soil specimens, the durability index tests were performed on each of the stabilized soil specimens with lime and alkaline activation. Figure 5 illustrates the results of the durability index versus cycles at different curing periods over the ultimate strength of soil without wetting/drying cycles. The results show that a significant decrease occurred in the strength and durability of the lime as the curing period increased compared to alkaline activation where an increment variance between 7 and 28 days during curing periods was observed to be insignificant before decrement occurred. This may be attributed to the process of a chemical reaction and the hardening process that occurs on lime and alkaline activation when increasing the curing periods, and thus a reduction in soil strength and durability index takes place. This behavior seems to coincidence with the result of unconfined compressive strength that has been explained before in Figure 3 and Figure 4 where the pattern shows the same increasing style until the third cycles before a decrease occurs.

**Figure 6:**
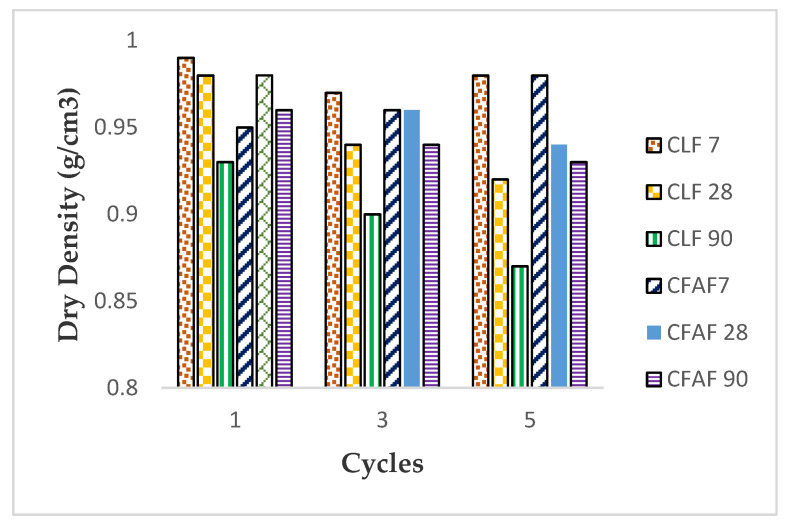
Dry density versus wetting/drying cycles.

**Figure 7:**
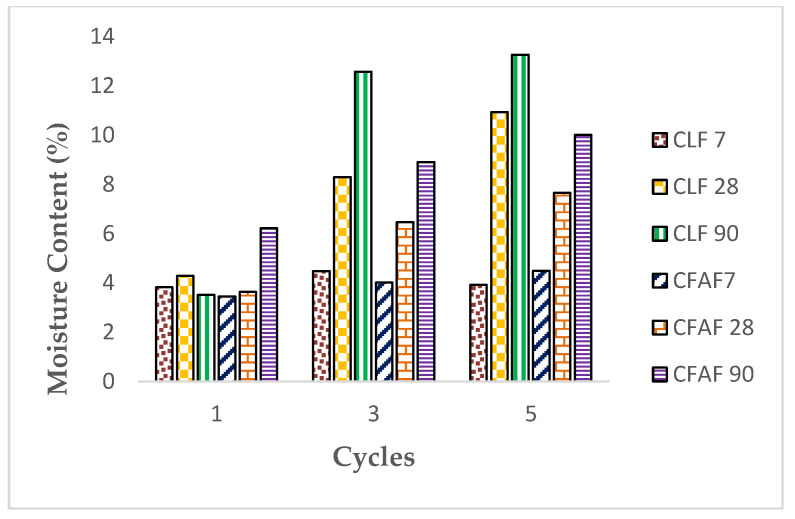
Moisture content versus wetting/drying cycles.

Figure 6 and Figure 7 represent the result of the dry density weight and moisture content of the mixture on the wetting/drying cycles. It can be seen that the wetting/drying cycles have a significant effect on the changes of the moisture content and dry density of the stabilized soil with lime and AA based on the three different curing periods.

It can be clearly seen that the dry density of the soil decreases slightly with the increase of wetting and drying cycles, and the water content increases with the increase of wetting/drying for all types of specimen, respectively. The decrement of the dry density is linked with the increase of the water content of the stabilized soil in which the percentage increase of the water content is in the range of range 2%–14%, increasing the cycle from the first cycle to the fifth cycle. The condition of increment of the water content on the stabilized soil is probably due to the fiber inclusion that helps in absorbing the water content in the soil at low dry density. Although there is a high water content in the soil with the increase of the curing period and wetting/drying cycles, the ability of fiber inclusion to help in retaining the water content while the stabilizer helps in reducing the dry density of the soil gives a good advantage in strengthening the soil. As for decreasing and increasing the dry density and water content for lime stabilization, inclusion of fiber in the soil matrix has helped in improving the water absorption rate, thus strengthening the soil properties. This statement has similar findings with past researchers [38,48,58,59].

**Figure 8:**
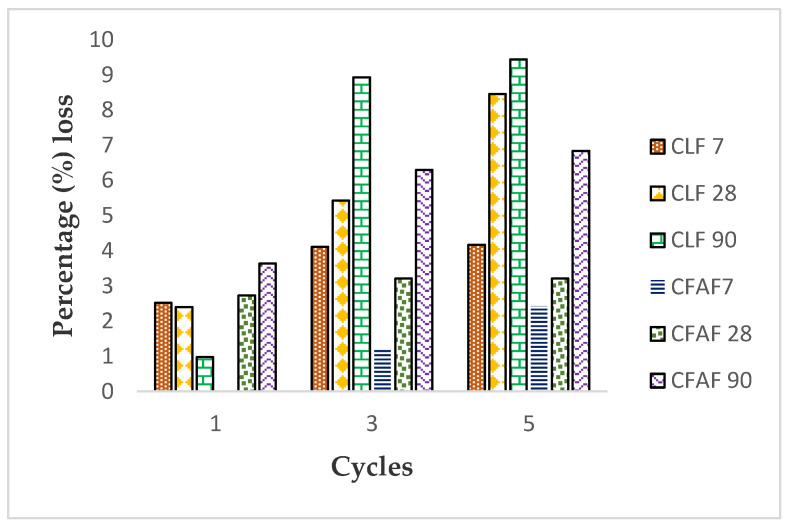
Percentage (%) of weight soil loss versus wetting/drying cycles.

Moreover, the increase of the water content for the soil specimen was believed to be due to the pozzolanic reaction that started to occur between the stabilizer and the activator. The process of wetting/drying of the specimen was disturbed as the sample started to react with the chemical. The percentage of water content and weight soil loss of the three curing periods of 7, 28, and 90 days for the lime and AA increase simultaneously in line with the increase in wetting and drying cycles, as can be seen in the results for Figure 7 and Figure 8, but it can be predicted that the dry density and the moisture content will be fluctuating throughout the incremental cycles for long-term conditions, respectively. However, the decreasing and increasing of the dry density and moisture content over the cycles are not too significant, but still affect the strength of the stabilized soil, as can be seen in Figure 3.

The percentage of weight loss for the soil stabilized with lime and AA versus the number of cycles is illustrated in Figure 8. The weight loss of soil stabilized with lime remarkably increases in the first cycles of wetting and drying and afterwards gradually increases as the cycles increase and curing periods with increment show a percentage loss of approximately 1% to 9.5%. It is noted that the stabilized soil with AA shows lower weight loss compared to the stabilized soil with lime with a percentage loss between 0% and 6.8% due to the stronger combination of alkaline activation with soil bonding structure. The gain of the percentage loss weight for the stabilized soil is due to the reaction of the cementitious reaction occurring in the soil (C-S-H and C-A-H), which is connected with the absorption of the water towards the voids in the soil. This statement is consistent with the findings by [60] for stabilized soil with granulated blast furnace slag (LAS) and lightweight Portland cement (LPC) at a variable soaking time with Na_2_SO_4_ solution. Equation (3) below shows the cementitious reaction that occurs when there is a high amount of Al_2_O_3_, SiO_2_, Fe_2_O_3_, and CaO with addition of water.

The hydration process is constructed as for CaO (lime) liberates OH- ions, as shown in Equation (4), which contributes in increasing the pH value up to 12. The pozzolanic reactions which are rich of Ca with the combination of silica (Si), alumina (Al), and iron (Fe) resulting for the C-A-H and C-S-H gels for longer curing stages [61,62] can be applied as shown in Equations (5) and (6).

6H_2_O + 2(3CaO·SiO_2_) = 3CaO·2SiO_2_·3H^2^O + 3Ca(OH)_2_, (3)

Hydrolysis: Ca(OH)_2_ = Ca ^++^ + 2(OH)_2_. (4)

Pozzolanic reactions:

Ca^++^ + 2(OH)^−^ + Al_2_O_3_ (pozzolan rich in alumina) = C-A-H, (5)

Ca^++^ + 2(OH)^−^ + SiO_2_ (pozzolan rich in silica) = C-S-H. (6)

### 3.3. Microstructural Analysis

**Figure 9:**
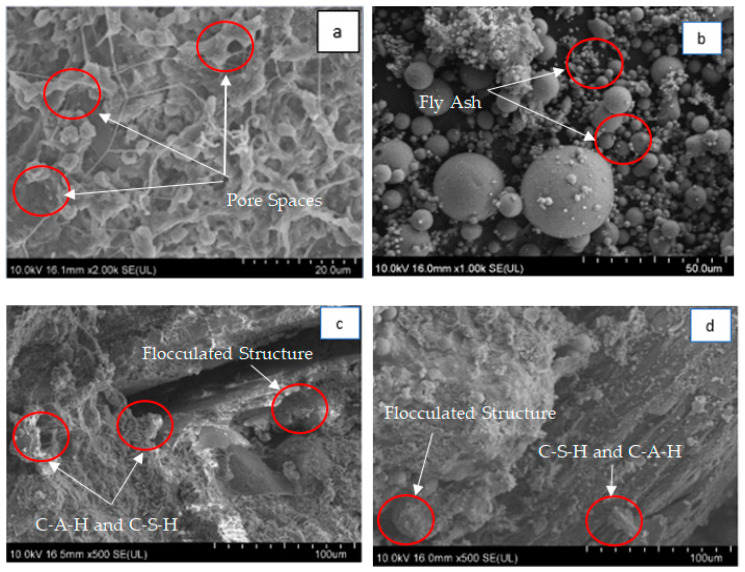
90 day curing period of the FESEM micrographs for (**a**) untreated clay, (**b**) fly ash, (**c**) CLF90 5C, and (**d**) CFAF90 5C.

Figure 9a–d shows the micrograph of untreated soil and treated soil with lime and AA samples for a curing period of 90 days. From Figure 9a it can be observed that the untreated soil specimen has a visible void with high porosity as well as a dispersed structure arrangement that is due to the high water content, which is similarly identified by [63], and a relatively loose structure around the “flakey bone particles”. According to [2], “intra-aggregate pores” were generated by the smaller pores that have been made up with the porosity. The “flakey particles” for the clay minerals usually appear associated with the montmorillonite group and are clearly visible as can be seen in Figure 9a. The dispersed structure is relatively brittle due to the high amount of water present in the clay soil, which loosens the strength of the soil particles. This contributed to an easy slide-over over each other when shear occurred on the clay particles and clay clusters, which attributed to the lowering of the strength and the stiffness of the clay soil [2,64].

Figure 9b,d depicts the micrographs of the treated soil specimens with lime and alkaline activation, respectively. It can be observed that the usage of the lime and AA have formed the particle into more flocculation upon the interaction that has been made with the clay and water/KOH. The micrograph further portrays the gaps that occurred among the soil particles that have been filled by the admixture that can be attributed to a dense soil with fewer pores on the soil structure.

**Figure 10:**
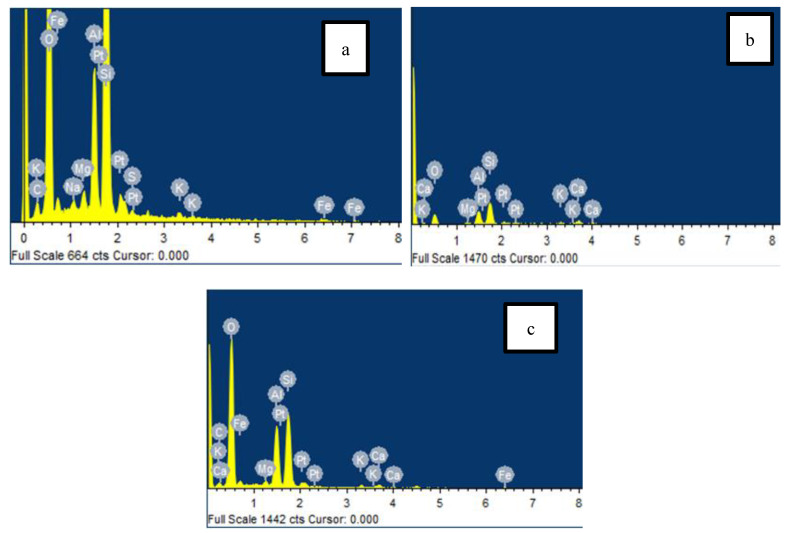
90 day curing period of the EDX spectrum for (**a**) untreated clay, (**b**) CLF 5C, and (**c**) CFAF90 5C.

Figure 10a–c shows the EDX spectrum of the FESEM analysis and Table 5 shows the soil element composition for the untreated and treated soil after wetting/drying cycles at a curing period of 90 days. From Table 5 and Figure 10b,c it can be observed that the percentage of Si and Al in the treated soil increased as the curing periods increased compared to the untreated clay soil (Figure 10a). It is confirmed that due to the high presence of Ca^2+^, SiO_2_, and Al_2_O_3_ in the specimens resulting from the lime and AA stabilizer the strength of the soil is increased. Furthermore, [65,66] has mentioned that the release of Ca from the stabilized material for the soaking process contributes in increasing the cementitious products. The pozzolanic reaction will continue as long as there is the availability of Ca, which results in the formation of calcium hydrates due to the presence of OH^−^, Ca^2+^, AlO_6_, and SiO_4_.

In addition, the cementation pozzolanic reaction of Calcium Aluminate Hydrate (C-A-H) and Calcium Silicate Hydrate (C-S-H) can be proven from Equations (5) and (6) above, where high pozzolan rich of silica and alumina contributes to the formation of CAH and CSH. Therefore, the formation of the binder has proven to contribute to the increment of unconfined compressive strength of the modified clay with lime and AA compared to the untreated clay soil due to the interlocking arrangement that occurred between the soil particles.

## 4. Conclusions

The findings on the research of the effect of the environmental condition, which subsequently are referred to wetting/drying cycles, on the durability and strength of the stabilized soil with lime and alkaline activation, can be concluded as follows:

- The effects of the wetting/drying test on the strength of the soil (for both stabilized lime and AA) for all different curing dates and cycles show a less significant fluctuation of increment and decrement except for a curing period of 90 days, where there is a sharp decrement that occurs at the first three cycles. This may be attributed to the effect of the wetting and drying that changes the formation of the soil occurring within the material with the increase of curing periods;
- The strength of treated soil with lime and AA started to decrease after a curing period of 28 days where the decrements were 13.19% and 38.64%, respectively, notable mostly at the fifth cycle. This is due to the interfacial bonding caused by a chemical reaction that occurred between the soil particles and the stabilizer being reduced, which resulted in loosening the soil particles as the cycle period increased. It is also due to the conditions where the specimens with dried conditions for a long-term duration were then exposed to a wetting/drying cycles, which tends to cause the soil to be in a moist condition, thus attributing to a decrease in soil strength;
- Dry density and water content versus cycles show a similar trend for both soils stabilized with lime and AA where decrement occurs for dry density and increment of the water content for all specimens, respectively. This may be due to the usage of the stabilizer and the fiber inclusion that helps in lowering the dry density of the soil and the ability of the stabilizer to absorb a large amount of water, as for lime and alkaline activation;
- An increasing trend of the soil percentage loss was observed with the increase of the wetting/drying cycles for both soil stabilization treatments with an accumulation of 3% to 5% increment, except for the first cycles of treatment with lime. This is due to the absorption of water into the soil voids that forms between the binders, thus leading to soil loss;
- Analysis of the FESEM and EDX results shows a dense and compacted soil with fewer visible pores after a curing period of 90 days. The admixtures help in filling the pores and strengthen the soil. From the EDX result, it is also confirmed that there is a formation of CSH and CAH (Figure 8) gels that contributes to the interlocking between the soil particles and thus strengthens the soil although there is some decrement and increment of the physical and mechanical properties of the soil.

## Figures and Tables

**Table 1:** Physical properties of marine clay soil.

Parameter	MC (%)	SG	LL (%)	PL (%)	OC (%)	pH	XRD
**Value**	72	2.57	52–72	32–40	6.88	7.5	Montmorillionite, illite, quartz, kaolinite, and halloysite

MC = moisture content, SG = specific gravity, LL = liquid limit, PL = plastic limit, OC = organic content, pH = pH meter.

**Table 2:** Properties of the coir fiber used in this study [17].

Coir Fiber	Value
**Length (cm)**	17–22
**Diameter (mm)**	0.18–0.38
**Tensile Strength (MPa)**	60–130
**Breaking Elongation (%)**	30
**Lignin (%)**	44
**Cellulose (%)**	48
**Ash (%)**	2
**Water Soluble (%)**	6

**Table 3:** Chemical composition of fly ash.

Chemical Composition	SiO_2_	Fe_2_O_3_	Al_2_O_3_	CaO
% by weight	57.47	4.71	15.37	3.32

**Table 4:** Admixture proportions of soil specimen.

Types of Soil	Designation Mix	Curing Period (Days)	Repetitive Sample	Number of Samples
C	Untreated clay soil	72,890	3	3 × 3 = 9
CLF	Clay, 5% of lime, 1% of fiber	72,890	3	3 × 3 = 9
CFAF	Clay, 60% of FA, 1% of fiber	72,890	3	3 × 3 = 9
Total No of Sample	27			

C = natural soil (clay), CLF = clay + lime + fiber (1%), FA = fly ash, CFAF = CFA + activator + fiber (1%).

**Table 5:** EDX Analysis for untreated and treated clay wetting/drying sample after a curing period of 90 days.

Mixture	Curing Period (Days)		Element (Wt %)						
		Si	Al	Ca	Mg	Na	C	O	Si/Al Al/Si
Clay	90	9.35	4.74	-	1.52	0.91	2.82	18.37	1.97 0.51
CLF	90	28.18	12.46	11.96	1.33	-	-	30.13	2.26 0.43
CFAF	90	22.15	6.55	3.27	0.74	-	9.43	43.58	3.37 0.29
